# Ionizing radiation alters functional neurotransmission in *Drosophila* larvae

**DOI:** 10.3389/fncel.2023.1151489

**Published:** 2023-07-06

**Authors:** Yi Zhang, Yihao Zhang, Cong Shen, Shun Hao, Wenlan Duan, Li Liu, Hongying Wei

**Affiliations:** ^1^North China Research Institute of Electro-Optics, Beijing, China; ^2^State Key Laboratory of Brain and Cognitive Science, CAS Center for Excellence in Biomacromolecules, Institute of Biophysics, Chinese Academy of Sciences, Beijing, China; ^3^College of Life Sciences, University of the Chinese Academy of Sciences, Beijing, China; ^4^China Electronics Technology Group Corporation No. 45 Research Institute, Beijing, China; ^5^CAS Key Laboratory of Mental Health, Institute of Psychology, Chinese Academy of Sciences, Beijing, China

**Keywords:** ionizing radiation, neurotransmission, dopamine, tyramine, PTTH neurons, *Drosophila* larvae

## Abstract

**Introduction:**

Patients undergoing cranial ionizing radiation therapy for brain malignancies are at increased risk of long-term neurocognitive decline, which is poorly understood and currently untreatable. Although the molecular pathogenesis has been intensively researched in many organisms, whether and how ionizing radiation alters functional neurotransmission remains unknown. This is the first study addressing physiological changes in neurotransmission after ionizing radiation exposure.

**Methods:**

To elucidate the cellular mechanisms of radiation damage, using calcium imaging, we analyzed the effects of ionizing radiation on the neurotransmitter-evoked responses of prothoracicotropic hormone (PTTH)-releasing neurons in *Drosophila* larvae, which play essential roles in normal larval development.

**Results:**

The neurotransmitters dopamine and tyramine decreased intracellular calcium levels of PTTH neurons in a dose-dependent manner. In gamma irradiated third-instar larvae, a dose of 25 Gy increased the sensitivity of PTTH neurons to dopamine and tyramine, and delayed development, possibly in response to abnormal functional neurotransmission. This irradiation level did not affect the viability and arborization of PTTH neurons and successful survival to adulthood. Exposure to a 40-Gy dose of gamma irradiation decreased the neurotransmitter sensitivity, physiological viability and axo-dendritic length of PTTH neurons. These serious damages led to substantial developmental delays and a precipitous reduction in the percentage of larvae that survived to adulthood. Our results demonstrate that gamma irradiation alters neurotransmitter-evoked responses, indicating synapses are vulnerable targets of ionizing radiation.

**Discussion:**

The current study provides new insights into ionizing radiation-induced disruption of physiological neurotransmitter signaling, which should be considered in preventive therapeutic interventions to reduce risks of neurological deficits after photon therapy.

## 1. Introduction

Cranial radiotherapy is widely used for treatment of brain tumors and effectively improves the survival of cancer patients. Unfortunately, long-term survivors routinely suffer serious neurological complications, including altered spatial recognition and deficits in learning and memory, attention and motor control (Armstrong et al., 2009). Individuals who receive radiation therapy treatment during childhood have a particularly increased incidence of many neurological and cognitive impairments (Ellenberg et al., 2009; Edelstein et al., 2011). Moreover, pathological studies have demonstrated that cranial radiation therapy is associated with deficits in neurogenesis and gliogenesis, neuroinflammation and decreased white matter integrity (Monje et al., 2002; Schuitema et al., 2013; Del Pilar et al., 2021). To reduce the neurotoxic side effects, understanding of the basic mechanisms underlying the neuronal dysfunction resulting from radiation urgently needs to be improved.

For decades, numerous studies have reported genetic damage produced by ionizing radiation (Carante and Ballarini, 2020; Beheshti et al., 2021; Cornforth et al., 2021). Ionizing radiation can affect nucleic acids, proteins, and complex lipids either by direct deposition of energy into macromolecules or by generating reactive oxygen species (ROS) including hydrogen peroxide, superoxide anions, and other free radicals (Azzam et al., 2012; Selim et al., 2016). Currently, DNA damage is known to have a strong impact on the pathogenesis of neurological diseases, including Alzheimer’s disease, Parkinson’s disease, and autism (McKinnon, 2017; Caldecott et al., 2022). Studies in the mouse hippocampus and cortex have shown that ionizing radiation immediately impairs the synaptic plasticity associated with molecular signaling pathways that are important for memory consolidation and learning behavior (Kempf et al., 2014, 2015). Furthermore, ionizing radiation-induced neuronal apoptosis has been extensively demonstrated in the developing neocortex (Nowak et al., 2006; Verreet et al., 2015). In recent years, radiation-induced cognitive dysfunction has been correlated with disorganization of neurons, which is accompanied by changes in neuronal migration, morphology, and brain structures (Selemon et al., 2013; Cacao et al., 2018; Puspitasari et al., 2020).

Neurotransmission enables signal transduction in neural circuitry and therefore is a major determinant of normal brain function. Many neuronal dysfunctions and brain disorders are linked to disruption of the neurotransmitter system (Kondziella, 2017; Brennenstuhl et al., 2019). An immunocytochemistry study showed that the number of glutamatergic and gamma-aminobutyric acid (GABA)ergic presynaptic terminals was altered in irradiated rats (Zhou and Roper, 2010). The neurotransmitter [serotonin, dopamine (DA), GABA] levels in whole brain tissue and the hippocampus were significantly decreased in mice exposed to ionizing radiation (Bekal et al., 2021). Moreover, blockage of N-methyl-D-aspartate-sensitive glutamate receptors attenuated radiation-induced learning deficits and apoptosis in immature neurons (Alaoui et al., 1995; Samari et al., 2013). Although neurotransmitters play an essential role in brain functions, to date, whether and how ionizing radiation alters postsynaptic neurotransmission is still unknown.

*Drosophila* has been widely employed to study neurotoxic damage and the function of radio-protective agents. Several studies showed that many important neurotoxic consequences of radiation exposure in mammals could be reasonably mimicked in *Drosophila* (Pandey and Nichols, 2011). Ionizing radiation induces DNA damage and neuron death in *Drosophila* (Kim et al., 2015; Sudmeier et al., 2015; Tanaka and Furuta, 2021). Ionizing radiation during the larval stage reduced brain size, delayed development, and reduced survival to adulthood (Paithankar et al., 2017; Wagle and Song, 2020). Furthermore, several studies have examined the effect of total radiation doses on developmental delays and survival in *Drosophila* (Halme et al., 2010; Sudmeier et al., 2015).

The goal of this study is to investigate ionizing radiation-induced deficits in neurotransmission. Previous studies in *Drosophila* larvae have demonstrated that ionizing radiation inhibited transcription of the prothoracicotropic hormone (PTTH) gene in PTTH-releasing neurons (Halme et al., 2010). PTTH is a neuropeptide in insects, analogous to the vertebrate adrenocorticotropic hormone (ACTH) (Ou et al., 2016). ACTH regulates the adrenal steroids in the vertebrate endocrine system (Miller, 2018). Similarly, PTTH promotes the production of steroid hormone ecdysone and metamorphosis of *Drosophila* larvae (McBrayer et al., 2007). PTTH is released from two pairs of neurons located specifically in the *pars lateralis* region, which along with the *pars intercerebralis* region, is considered homologous to the mammalian hypothalamus (Hückesfeld et al., 2021). The activity of PTTH-releasing neurons is modulated by diverse neurotransmitter signals, which are necessary for successful metamorphosis and survival during *Drosophila* larval development. DA and tyramine (TA) both elicit inhibitory responses of PTTH neurons (Hao et al., 2021). DA receptor signaling via D1- or D2-like receptors is highly conserved between *Drosophila* and human. Activation of D1- or D2-like receptors could increase or decrease intracellular cyclic adenosine monophosphate (cAMP) levels in both species, respectively. Additionally, a non-canonical DA/ecdysone receptor (DopEcR) has been classified in *Drosophila* (Karam et al., 2020). TA is considered to be the homology of norepinephrine in vertebrates. Three classes of G-protein coupled TAR receptors (TAR1–3) exist in *Drosophila*. TA inhibits adenylyl cyclase and decreases cAMP levels via TAR1 receptors. Activation of TAR2 and TAR3 is associated with increases of intracellular calcium levels (Finetti et al., 2021). Here, we determined whether ionizing radiation could alter neurotransmission in postsynaptic PTTH neurons, with a companied abnormal development of *Drosophila*.

To elucidate the cellular and synaptic mechanisms of radiation damage, we analyzed the effects of ionizing radiation on the neurotransmitter-evoked responses of PTTH neurons. We characterized the dose-response functions for the inhibitory effects of DA and TA in PTTH neurons. Surprisingly, irradiation with 25 Gy of gamma radiation resulted in increased DA and TA sensitivity without compromising the viability and axo-dendritic architecture of PTTH neurons. In contrast, irradiation with 40 Gy of gamma radiation decreased DA and TA sensitivity, physiological viability and the arborizations of PTTH neurons. These physiological and anatomical changes could affect the development of *Drosophila* larvae. Exposure to these two radiation doses (25 Gy and 40 Gy) are within the recommended range for radiotherapy against tumors (Hellevik and Martinez-Zubiaurre, 2014; Trifiletti et al., 2020). This study provides, for the first time, convincing evidence for marked alterations of functional neurotransmission after exposure to ionizing radiation. These results could reveal the possible underlying mechanisms of radiation-induced neuronal dysfunction and enable development of improved defensive strategies.

## 2. Materials and methods

### 2.1. *Drosophila* stocks and gamma irradiation

The fly strains were reared with a standard medium at 24°C and 60% relative humidity under a light-dark cycle of 12 h:12 h, as described by Guo et al. (1996). For calcium imaging and developmental experiments, flies of the PTTH-Gal4 (obtained from Christian Wegener’s lab, University of Würzburg) were crossed with flies of the UAS-GCaMP6m strain. Crosses of the line PTTH-Gal4 with flies of the UAS-Epac-camps (BDSC#25408) were used for cAMP imaging experiments. In calcium imaging experiments combined with TA receptor RNAi, the homozygous flies (UAS-GCaMP6m; PTTH-Gal4) were crossed with TAR1 RNAi fly stocks (THU2969, Tsinghua *Drosophila* stock center). Third instar larvae expressing a mouse CD8-tagged green fluorescent protein (GFP) fusion protein (UAS-mCD8::GFP, BDSC#5137) under the control of PTTH-Gal4 driver were used for visualization of PTTH neurons. In optogenetic experiments, flies of the PTTH-Gal4 were crossed with flies carrying UAS-CsChrimson (BDSC#82181) or UAS-GtACR1 (BDSC#92983), respectively, to drive the expression of channelrhodopsins in the experimental groups. The three strains were crossed with *w*^1118^ flies to serve as the control groups.

At 92 h after egg deposition (AED), vials containing third-instar larvae were placed on a rotating plate and exposed to a single dose of gamma-radiations (25 Gy or 40 Gy) with a dose rate of 2.5 Gy/min in a gamma chamber (^60^Co as gamma source at the National Institute of Metrology of China). Control vials were handled identically but without radiation exposure.

### 2.2. Calcium imaging and cAMP imaging

At 120–176 h AED, the larvae brains were dissected and placed in a recording chamber on the stage of a Zeiss LSM 880 confocal microscope system. Calcium- and cAMP imaging recordings were performed during continuous superfusion with standard larval extracellular Ringer solution (128 mM NaCl, 2 mM KCl, 4 mM MgCl_2_, 1.5 mM CaCl_2_, 35 mM Sucrose, 5 mM Trehalose, 5 mM HEPES, pH 7.2) or stimulus presentation (1 ml/min). To monitor calcium or cAMP changes, the fluorescence intensities of genetically encoded calcium indicator GCaMP6m or cAMP biosensor Epac-camps in PTTH neurons were analyzed, respectively. The cell bodies of individual PTTH neurons were manually defined for the regions of interest. In calcium imaging experiments, the GCaMP6m was excited using a 488-nm laser at 3 Hz. The average fluorescence intensity (emission signal from 508 to 535 nm) within each region of interest was calculated. The fluorescence signal of GCaMP6m, which reflects the intracellular calcium levels of PTTH neurons, was calculated as the fluorescence change divided by resting fluorescence (ΔF/F_0_) for each frame to create a response trace as the function of time. In cAMP Imaging experiments, a 458 nm laser was used to excite Epac-camps in PTTH neurons. The emission signals of cyan and yellow fluorescent protein (460–520 nm for cyan fluorescent protein; 520–580 nm for yellow fluorescent protein) were separated by a Carl Zeiss confocal splitter. The ratio of cyan and yellow fluorescent protein ratio was calculated for each frame, which reflects the intracellular cAMP level of PTTH neurons. The background was subtracted from each image before calculation of the ratio.

For dose-response experiments, the DA and TA dissolved in Ringer solution were bath applied for 2 min at different physiological relevant concentrations (100 nm–1 mM). The brains were continuously superfused with Ringer solution except stimulus application. During stimulus presentation bath Ringer solution was replaced by the respective stimulus solution. To examine the dose-dependence of DA and TA responses, the amplitudes of calcium responses were normalized to those obtained with 500 μM DA or TA (= 100%), respectively. Half-maximal inhibitory concentrations (IC_50_) of neurotransmitters were calculated from normalized dose-response curves by fitting a standard 4-parameter logistic model. The slope of dose-response curve was examined. To analyze the TA receptors, TAR1 receptor antagonist yohimbine was used in calcium imaging experiments. The brains were incubated with 100 μM yohimbine before being treated with Ringer solution containing 100 μM TA and the same antagonist. In cAMP Imaging experiments, the enzyme adenylyl cyclase activator forskolin (10 μM) were applied to increase the intracellular cAMP concentration before TA treatment. The combined effect of TA and forskolin was examined. During the viability-test, Ringer solution containing 20 mM KCl was used to stimulate the living neurons.

### 2.3. Measurements of axo-dendritic length in PTTH neurons

For the visualization and morphological analysis of PTTH neurons, the brains and prothoracic glands (PG) of wandering third instar larvae were dissected and fixed in 4% paraformaldehyde for 30 min at room temperature. Afterward, the brain and PG complexes were washed 3 times for 30 min with phosphate buffered saline containing 0.3% Triton X-100. Finally, they were mounted in Vectashield Fluorescent Mounting Media (Vector Laboratories) between two coverslips using two spacers (Zweckform, Oberlaindern) to prevent compressions. The brain and PG complexes were scanned with a confocal microscope (Zeiss LSM 880). The acquired images were then processed with Amira 5.3.3 software. The axo-dendritic lengths of GFP-labeled PTTH neurons were measured with the auto-skeleton module in Amira.

### 2.4. Developmental experiments

For optogenetic experiments, 100 pairs of female and male flies were collected and crossed in a fresh food bottle for 3 days to maximize the fecundity. Homogeneous eggs of indicated genotypes were collected on apple juice agar plates with yeast paste within 4 h. After 24 h, newly ecdysed first-instar larvae were transferred onto a food plate containing 0.4 mM all-trans-retinal. They were allowed to feed and develop for 2 days in darkness. Then the morphological characteristics of larvae were monitored to synchronize after molting from second to third-instar larvae stage. Within a period of 4 h, freshly ecdysed third-instar larvae of similar sizes were transferred to a new food plate containing 0.4 mM all-trans-retinal and reared in darkness for 12 h. Afterward, optogenetic manipulation was performed by exposing the plates to red or green light. Red LEDs of 600 nm (25 μW/mm^2^) and green LEDs of 520 nm (30 μW/mm^2^) were used to activate CsChrimson-expressing and inhibit GtACR1-expressing PTTH neurons, respectively. Thereafter, the number of pupae were counted every 3 h, 6 times each day. Pupariation ratio was the cumulative pupae number of each time point as a percentage of total pupae number. Using a logistic curve fitting model, we calculated the time at which the 50% pupariation ratio was observed.

For irradiation experiments, 100 pairs of mated flies were allowed to oviposit in a new culture vial for 4 h to obtain homogeneous samples. At 92 h AED, larvae vials were divided in three groups: the first was irradiated with 25 Gy, the second with 40 Gy, and the third group was used as control (0 Gy). Thereafter, the irradiated and control larvae were then allowed to complete development under standard rearing conditions. For developmental timing analysis, the number of pupae were counted at two regular time points of each day with an interval of 8 h. The pupariation timing curve and the time at which the 50% pupariation ratio occurred were determined. Eclosion rate was the total adult flies as a percentage of total pupae number.

### 2.5. Statistical analyses

Statistical analysis was performed using SPSS 23.0 statistical software and GraphPad Prism 6.0. Data were given as mean ± standard error of the mean. Shapiro–Wilk test was used for normality test. One-way ANOVA was applied to analyze the mean difference among normally distributed data groups. Homogeneity of variance test was performed. If equal variances could be assumed, LSD was used for pairwise statistical comparisons, otherwise Tamhame was performed. If the data did not show normal distribution, the Mann–Whitney test was employed for comparing two groups, and the Kruskal–Wallis test with Dunn’s post-hoc test was used for multiple comparisons. For RNAi experiments, unpaired *t*-test was performed to compare the normally distributed calcium responses between TAR1 RNAi and *w*^1118^ control groups. In pharmacological experiments, calcium responses of the same cells in *w*^1118^ control group before and after blocker treatment were compared by using the Wilcoxon signed rank test, since the data were not normally distributed. In cAMP imaging experiments, paired *t*-test was used to compare normally distributed cAMP levels after vehicle and TA applications. According to the *P*-values significant and non-significant differences were shown in figures as asterisks or ns (*p < 0.05; **p < 0.01; ***p < 0.001; ns, not significant *p* > 0.05).

## 3. Results

### 3.1. Ionizing radiation alters DA responses in PTTH neurons

**FIGURE 1:**
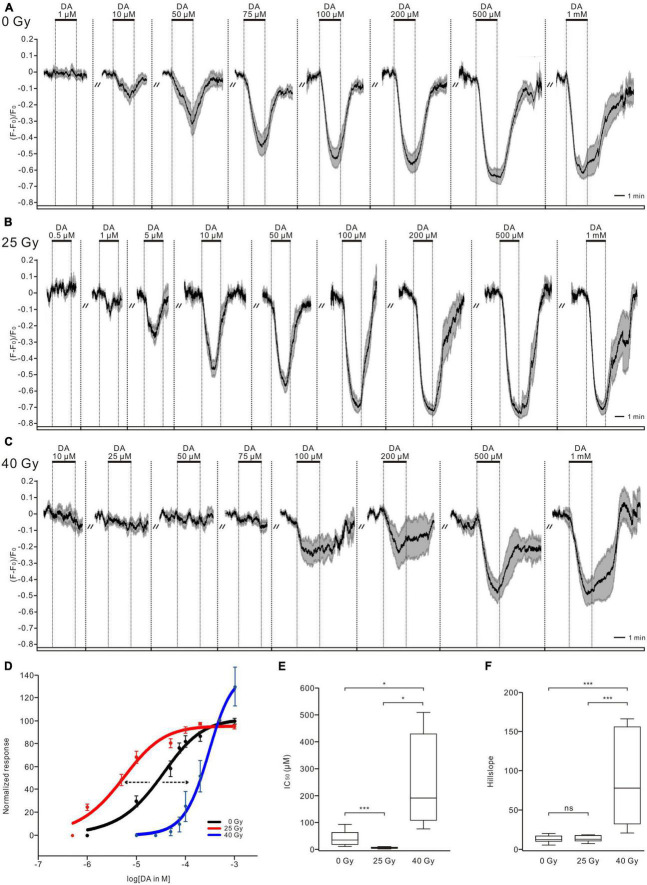
Ionizing radiation affects dopamine (DA) sensitivity of PTTH neurons. **(A–C)** DA application decreased intracellular calcium levels of PTTH neurons dose-dependently in unirradiated larvae (*n* = 10, 0 Gy) **(A)**, larvae exposed to doses of 25 Gy (*n* = 9) **(B)** and 40 Gy (*n* = 8) **(C)** gamma radiation. Response traces were drawn with the mean ± SEM. **(D)** Normalized dose-response curves of PTTH neurons in response to DA applications. As compared to control group (black, 0 Gy), the DA dose-response curve in the 25-Gy radiation group (red) was shifted to the left, and that in the 40-Gy radiation group (blue) was translated to the right, indicating an increased and decreased sensitivity, respectively. **(E)** The IC_50_ values in the 25-Gy group (*n* = 9) were significantly lower, and in the 40-Gy group (*n* = 8) significantly higher as compared to control group (*n* = 10) (ANOVA Tamhane test). **(F)** The slope values of DA dose-response curves in the 25-Gy group (*n* = 9) and control group (*n* = 10) showed no significant difference. In contrast, the slope values in the 40-Gy group (*n* = 8) were significantly higher than those in the 25-Gy or control group (Kruskal–Wallis test). *p < 0.05, ***p < 0.001; ns, not significant *p* > 0.05.

To examine physiological activity, the somatic calcium signals from GCaMP6m-expressing PTTH neurons were recorded. DA application triggered dose-dependent decreases in the intracellular calcium concentration of PTTH neurons in the control and radiation groups (larvae exposed to 25 and 40-Gy doses of gamma irradiation). After DA washout from the bath solution, the intracellular calcium levels returned to the control levels (Figures 1A–C). The threshold concentration for the effect of DA varied between 10 and 50 μM in the control (0 Gy) group (*n* = 10) (Figure 1A). An inhibitory effect of 1 μM DA on the intracellular calcium concentration of PTTH neurons in larvae exposed to 25 Gy of radiation was observed in all tested larvae brains (*n* = 9) (Figure 1B). The PTTH neurons in the 40-Gy group showed a higher threshold concentration of 75–200 μM DA (*n* = 8) (Figure 1C). To assess the radiation-induced changes in responses of PTTH neurons to DA application, we constructed dose-response curves and compared both the IC_50_ and slope values among the three groups (Figures 1D–F). Gamma irradiation with doses of 25 and 40 Gy shifted the dose-response curves to the left and right, respectively (Figure 1D). Compared with the unirradiated control group (0 Gy), the IC_50_ in the 25-Gy radiation group was significantly decreased (7.44 ± 2.14 μM in the 25-Gy group, *n* = 9; 41.37 ± 8.52 μM in the control group, *n* = 10) (Figure 1E). The DA dose-response curve slope was not significantly different between the 25-Gy radiation group and the control group (Figure 1F). Therefore, irradiation with 25 Gy of gamma radiation resulted in a parallel leftward shift of the dose-response curve in response to DA application. In contrast, the IC_50_ (247.43 ± 58.79 μM) and slope of the DA dose-response curve in the 40-Gy group (*n* = 8) were significantly higher than those of the 25-Gy group or control group (Figures 1E, F), indicating a right-shifted dose-response curve with a reduced dynamic range. These results demonstrated that 25 Gy of radiation increased, whereas 40-Gy exposure decreased, the sensitivity of PTTH neurons to DA application.

### 3.2. Ionizing radiation alters TA responses in PTTH neurons

**FIGURE 2:**
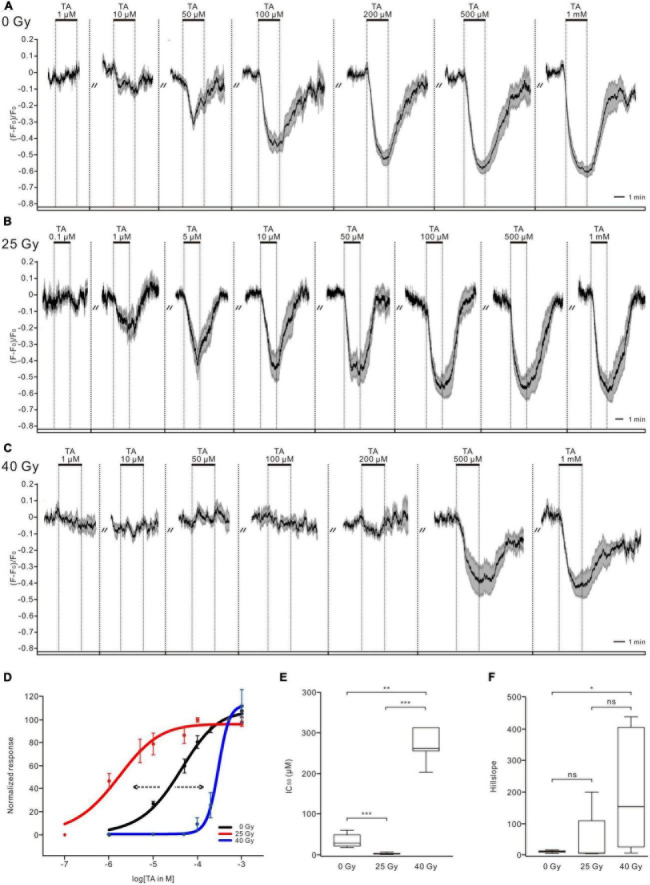
Ionizing radiation alters tyramine (TA) sensitivity of PTTH neurons. **(A–C)** TA application induced calcium decreases in PTTH neurons dose-dependently in the control larvae group (*n* = 9, 0 Gy), 25-Gy radiation group (*n* = 8) **(B)** and 40-Gy radiation group (*n* = 7) **(C)**. Response traces were drawn with the mean ± SEM. **(D)** Normalized TA dose-response curves of PTTH neurons. Compared with control group (black), the 25 and 40-Gy radiation exposure caused a leftward (red) and rightward (blue) shift of dose-response curve, respectively. **(E)** The IC_50_ values in the 25-Gy group (*n* = 8) were significantly lower, and in the 40-Gy group (*n* = 7) significantly higher as compared to the control group (*n* = 9) (Kruskal–Wallis test). **(F)** The slope values in the 40-Gy group were significantly higher than those in the control group. The slope values in the 25-Gy and the control group showed no significant difference (Kruskal–Wallis test). *p < 0.05; **p < 0.01; ***p < 0.001; ns, not significant *p* > 0.05.

The TA treatment decreased calcium levels of PTTH neurons in a dose-dependent manner in the non-irradiated control group (*n* = 9, Figure 2A), in the 25-Gy (*n* = 8, Figure 2B) and 40-Gy radiation groups (*n* = 7, Figure 2C). In larvae irradiated with a 25-Gy dose of radiation, TA (1 μM) induced a decrease in intracellular calcium. The magnitude of the peak response was dose-dependent between 1 and 100 μM (*n* = 8, Figure 2B). The TA dose-response curve in the 25-Gy group was shifted to the left compared with that in the control group (Figure 2D). A 25-Gy exposure led to a significantly decreased IC_50_ without affecting the slope, indicating an increase in TA sensitivity (IC_50_ = 5.67 ± 3.32 μM for the 25-Gy group, *n* = 8; IC_50_ = 45.9 ± 16.39 μM for the control group, *n* = 9) (Figures 2E, F). In larvae exposed to 40 Gy of radiation, higher concentrations of TA treatment (200 μM − 1 mM) led to decreases in calcium level. In contrast, the application of lower concentrations, within more common physiological ranges (1 − 100 μM), could not induce calcium responses of PTTH neurons (*n* = 7, Figure 2C). The TA dose-response curve was shifted to the right compared with that in the control group (Figure 2D). The IC_50_ (448.11 ± 189.44 μM) and slope values in the 40-Gy group were significantly higher than those in the control group (Figures 2E, F). These results indicated that 25 and 40 Gy of gamma radiation could increase and decrease the sensitivity of PTTH neurons to TA application, respectively.

### 3.3. TA decreases intracellular calcium and cAMP levels in PTTH neurons via TAR1-type receptors

**FIGURE 3:**
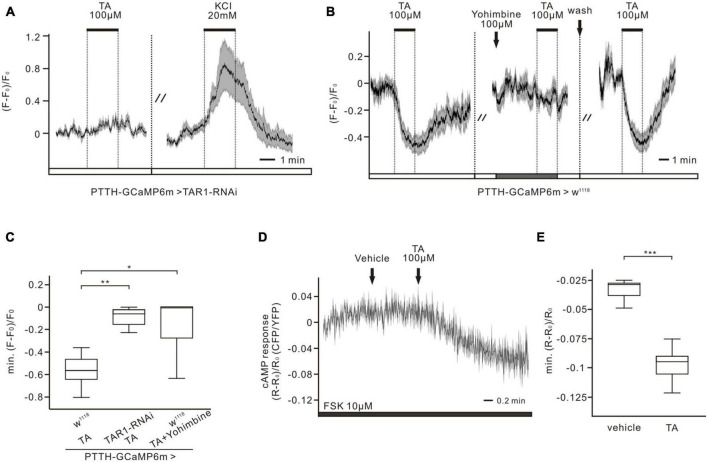
Tyramine (TA) elicited decreases of calcium and cAMP levels are conveyed by TAR1-type receptors in PTTH neurons. **(A,B)** Ca^2+^ responses of the RNAi experimental group (co-expressing GCaMP6m and TAR1 RNAi in PTTH neurons) and the *w*^1118^ control group to 100 μM TA. **(A)** When downregulating the expression of TAR1 receptors specifically in PTTH neurons, no TA responses could be detected. The activity of PTTH neurons was demonstrated to be normal by the application of 20 mM KCl (*n* = 7). **(B)** 100 μM TAR1 receptors antagonist yohimbine was able to completely block the response of PTTH neurons to 100 μM TA (*n* = 8). **(C)** Statistical analysis of the Ca^2+^ responses to 100 μM TA in experimental and control group. Calcium responses were significantly reduced, when downregulating the expression level of TAR1 receptors (unpaired *t*-test) (*n* = 7) or blocking TAR1 receptors with antagonist yohimbine (Wilcoxon signed ranks test) (*n* = 8). **(D,E)** Forskolin (FSK, 10 μM) was perfused to the brains, which could cause an increase in cAMP concentration. TA application (100 μM), but not vehicle control, could significantly decrease intracellular cAMP levels (paired *t*-test) (*n* = 7). *p < 0.05; **p < 0.01; ***p < 0.001.

To identify the responsible TA receptors in PTTH neurons, RNAi and pharmacological experiments were performed in combination with calcium imaging. Three classes of G-protein coupled TAR receptors exist, TAR1–3, which are responsible for mediating TA-dependent intracellular pathways in *Drosophila*. TAR1 is mainly associated with inhibition of adenylyl cyclase activity and decreased cAMP levels. Activation of TAR2 and TAR3 increases intracellular calcium levels (Finetti et al., 2021), which is inconsistent with the calcium response of PTTH neurons to TA application (Figure 2A). Thus, we investigated the impacts of TAR1 knockdown in PTTH neurons on the TA responses. When the expression of TAR1 in PTTH neurons was downregulated by RNAi, the intracellular calcium levels remained close to baseline after perfusion with 100 μM TA (*n* = 7, Figure 3A). TA application induced the calcium decreases of PTTH neurons in the *w*^1118^ control group, which were blocked by pre-incubation with the TAR1 antagonist yohimbine (100 μM) (*n* = 8, Figure 3B). Statistical analysis revealed that the TA responses were significantly reduced in both the RNAi experimental group (*n* = 7) and the control group incubated with yohimbine (*n* = 8), compared to those in the *w*^1118^ control group without yohimbine (*n* = 8, Figure 3C). Moreover, to detect TA-induced cAMP decreases, forskolin (10 μM) was perfused into the brain, which could directly activate adenylyl cyclase and increase intracellular cAMP levels in PTTH neurons. Bath application of 100 μM TA significantly decreased forskolin-stimulated intracellular cAMP levels, whereas vehicle application in the bath did not affect cAMP levels (*n* = 7, Figures 3D, E). Therefore, TA-induced cAMP and calcium decreases in PTTH neurons were mediated via TAR1-type TA receptors.

### 3.4. Ionizing radiation with 40 Gy, but not 25 Gy of gamma radiation, affects the physiological viability and axo-dendritic length of PTTH neurons

**FIGURE 4:**
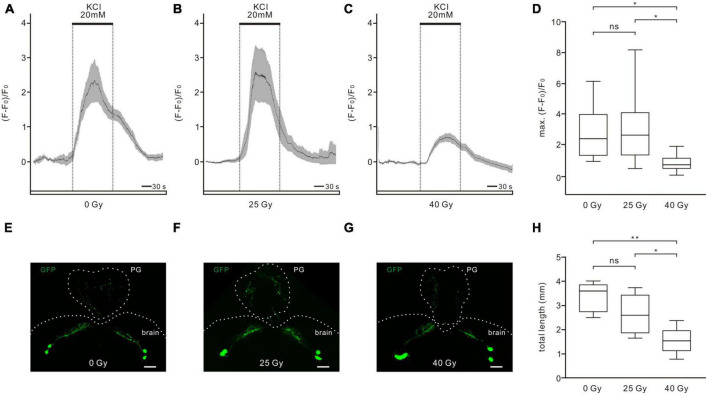
Effects of ionizing radiation on physiological viability and axon-dendritic length of PTTH neurons. **(A–C)** Calcium increases in response to 20 mM KCl application could be observed in all tested PTTH neurons in the control (A, 0 Gy), 25-Gy **(B)** and 40-Gy **(C)** radiation group. Response traces were drawn with the mean ± SEM. **(D)** There was no significant difference of the maximum responses between the control group (*n* = 9) and the 25-Gy radiation group (*n* = 10). The maximum calcium response was significantly reduced in the 40-Gy radiation group (*n* = 16) (ANOVA, Tamhane test). **(E–G)** Dendritic and axon arborizations of PTTH neurons in the control **(E)**, 25-Gy **(F)** and 40-Gy **(G)** radiation group. The PTTH fibers were observed in the brain and prothoracic gland (PG). Scale bars, 40 μm. **(H)** The length of GFP-labeled axo-dendritic branches did not differ significantly in PTTH neurons in the control (*n* = 5) and 25-Gy radiation group (*n* = 5). PTTH neurons in the 40-Gy radiation group (*n* = 5) had a significantly shorter length than those in the control and 25-Gy radiation group (ANOVA, LSD test). *p < 0.05; **p < 0.01; ns, not significant *p* > 0.05.

To examine the physiological viability of PTTH neurons after 25 and 40 Gy of ionizing radiation exposure, changes in intracellular calcium levels in response to Ringer solution containing 20 mM KCl were examined (Figures 4A–D). A high K^+^ concentration depolarizes the cell membranes, which can promote a depolarization-dependent calcium influx in physiologically intact neurons. However, compromised neuronal viability impairs KCl-induced physiological responses. Application of 20 mM KCl increased calcium levels in PTTH neurons in the control group (Figure 4A), 25-Gy radiation group (Figure 4B), and 40-Gy radiation group (Figure 4C). The calcium response amplitude was not significantly different between the 25-Gy radiation group (*n* = 10) and the control group (*n* = 9). However, the calcium increase in the 40-Gy radiation group (*n* = 16) was significantly smaller than those in the 25-Gy radiation group and control group (Figure 4D). Therefore, 25 Gy of gamma irradiation did not affect voltage-dependent physiological responses which represent neuronal viability. A dose of 40 Gy of radiation resulted in reduced viability of PTTH neurons.

To analyze whether gamma irradiation could change the morphology of PTTH neurons, the total length of GFP-labeled fibers in PTTH neurons was measured (Figures 4E–H). In all control and radiation groups, two prominent neurons could be observed in each hemisphere of the central brain. These PTTH neurons are unipolar and prominently project their axons to the PG (Figures 4E–G). Some axonal side branches terminate in the central brain. The dendritic arborizations extend toward the same direction as the axons (McBrayer et al., 2007). The total length of GFP-labeled fibers did not show a significant difference between PTTH neurons in the control (3,361.32 ± 275.66 μm, *n* = 5) and the 25-Gy group (2,641.22 ± 374.18 μm, *n* = 5). However, larvae exposed to 40 Gy of gamma radiation exhibited a significantly shorter length of PTTH fibers (1,544.29 ± 255.09 μm, *n* = 5), compared to those without radiation exposure or with 25-Gy radiation exposure (Figure 4H). These results demonstrated that gamma irradiation with a dose of 40 Gy, but not 25 Gy, changed the axo-dendritic length of PTTH neurons.

### 3.5. Manipulating activity of PTTH neurons affects the development of *Drosophila* larvae

**FIGURE 5:**
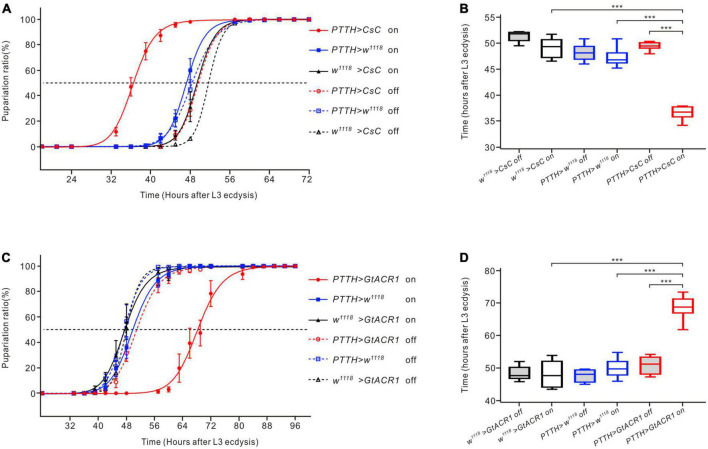
Effects of optogenetic activation and inhibition of PTTH neurons on the development of *Drosophila* larvae. **(A)** Pupariation timing curves of *Drosophila* larvae. Activation of PTTH neurons expressing CsChrimson (CsC) under red LEDs shifted the curve to the left compared to the control groups. **(B)** The time of 50% pupariation ratio in the experimental group (*n* = 6) was significantly shorter than that in the control groups (*n* = 6) (ANOVA, LSD test). **(C)** Pupariation timing curves of *Drosophila* larvae. Inhibition of PTTH neurons expressing GtACR1 under green LEDs shifted the curve to the right compared to the control groups. **(D)** The time of 50% pupariation ratio in the experimental group (*n* = 6) was significantly longer than that in the control groups (*n* = 6) (ANOVA, LSD test). ***p < 0.001.

To investigate whether modulating the activity of PTTH neurons could alter the developmental timing of *Drosophila* larvae, channelrhodopsins were expressed in PTTH neurons to activate or inhibit them under specific light conditions. Compared to the control groups, activation of CsChrimson-expressing PTTH neurons using red light led to a leftward shift of the pupariation timing curve (Figure 5A). The timing at which the 50% pupariation ratio occurred was significantly accelerated by approximately 12 h (Figure 5B). In contrast, inhibition of GtACR1-expressing PTTH neurons with green light resulted in a rightward shift of the pupariation timing curve (Figure 5C). The timing at 50% pupariation ratio was significantly delayed (∼20 h) compared to the control groups (Figure 5D). These results indicated that optogenetically modulating the neuronal activity of PTTH neurons changed the timing of developmental transitions in *Drosophila* larvae.

### 3.6. Ionizing radiation affects the development of *Drosophila* larvae

**FIGURE 6:**
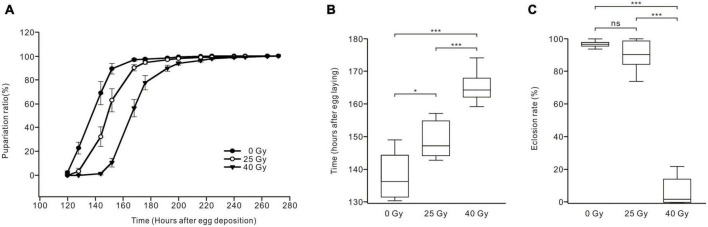
Effects of ionizing radiation on the development and survival of *Drosophila* larvae to adulthood. **(A)** Pupariation timing curves of *Drosophila* larvae. Radiation exposure right shifted the curve as compared to control group (solid dots), with the 40-Gy group (solid triangles) showing a greater shift than the 25-Gy group (hollow dots). **(B)** The time of 50% pupariation ratio in the 25-Gy group (*n* = 8) was significantly longer than that of the control group (*n* = 7). The larvae in the 40-Gy group (*n* = 5) exhibited significantly longer development than the control and 25-Gy group (ANOVA, LSD test). **(C)** The eclosion rate of larvae in three groups. There was no significant difference between the control group (*n* = 10) and the 25-Gy group (*n* = 11). However, the eclosion rate was significantly reduced in the 40-Gy group (*n* = 6) compared with the control group or 25-Gy group (ANOVA, Tamhane test). *p < 0.05, ***p < 0.001; ns, not significant *p* > 0.05.

To determine whether the development and metamorphosis of *Drosophila* larvae are affected after ionizing radiation exposure, the pupariation timing curve and eclosion rate were analyzed (Figure 6). Compared with the control group, gamma irradiation with doses of 25 Gy and 40 Gy shifted the pupariation timing curve to the right, with the 40-Gy group showing a greater shift than the 25-Gy group (Figure 6A). The time at which the 50% pupariation ratio was observed increased significantly from 138.06 ± 2.9 h AED for larvae in the control group (*n* = 7) to 148.88 ± 2.06 h AED for the 25-Gy radiation group (*n* = 8) and 165.36 ± 2.58 h AED for the 40-Gy radiation group (*n* = 5), indicating developmental delays increased with a higher radiation dose (Figure 6B). Most larvae successfully eclosed in the control group (96.75% ± 0.05%, *n* = 10) and the 25-Gy radiation group (90.02% ± 2.73%, *n* = 11). However, the eclosion rate of the 40-Gy radiation group (6.73% ± 3.8%, *n* = 6) was significantly lower than those of the control and 25-Gy radiation groups (Figure 6C). Thus, both 25 Gy and 40 Gy of gamma irradiation delayed the pupariation of *Drosophila* larvae, with 40 Gy producing a longer delay. Moreover, 40 Gy of radiation gravely reduced the survival of *Drosophila* larvae to adulthood while 25 Gy had little effect.

## 4. Discussion

In the present study, we examined the effects of ionizing radiation on functional neurotransmission in PTTH neurons, which are centrally important for *Drosophila* larvae development. The key findings are as follows: (1) 25 Gy of gamma irradiation resulted in increased sensitivity of PTTH neurons to DA and TA application, without alterations in neuronal viability and excitability. (2) A higher dose of gamma irradiation (40 Gy) damaged the neurons and decreased the DA and TA sensitivity of PTTH neurons. Therefore, we have clearly demonstrated, for the first time, ionizing radiation-induced alterations in functional neurotransmission, which could be a contributing risk factor to neurological diseases.

*Drosophila* has served as an excellent model for investigating many human nervous system functions and disorders because of its powerful genetics, relatively simple nervous system, and highly conserved neuroendocrine system and neurological pathways (Pandey and Nichols, 2011; Rudrapatna et al., 2012; Cunliffe et al., 2015). To date, several studies have used *Drosophila* to study the neurotoxic consequences of radiation exposure and radioprotective agents, which have potential for clinical application (González et al., 2018; Paithankar et al., 2018). Exposing third-instar larvae to ionizing radiation dose-dependently caused delays in development, reduced survival to adulthood, shortened the adult lifespan, and increased cell death in the adult brain (Sudmeier et al., 2015). During *Drosophila* pupariation and metamorphosis, continued neurogenesis occurs for approximately 2–3 days. The larval metamorphosis is triggered by a rapid increase in the level of ecdysone, an essential insect steroid hormone. The neuropeptide PTTH promotes ecdysone biosynthesis and the larval-to-pupae transition. Appropriate PTTH expression and release control the timing of development (McBrayer et al., 2007). Here, developmental experiments combined with optogenetic manipulation of the neuronal activity demonstrated that activating PTTH neurons accelerated, while inhibiting them delayed the development. These results suggested the physiological activities of PTTH neurons could influence and represent PTTH production and release, and therefore regulate the timing of developmental transitions. Furthermore, we examined the development of *Drosophila* larvae irradiated at 92 h AED. Ionizing radiation with both 25 and 40-Gy doses significantly delayed pupariation timing, which could be caused by a decrease in PTTH release. Our earlier study showed that PTTH neuron activity could be modulated by diverse neurotransmitter signaling pathways in *Drosophila* larvae, including DA and TA pathways (Hao et al., 2021). We therefore hypothesized that ionizing radiation could induce alterations in the effects of neurotransmitters on PTTH neurons, which play an essential role in larval development and survival. Surprisingly, we found opposite effects of two doses of gamma irradiation on the neurotransmitter sensitivity of PTTH neurons. A lower dose (25 Gy) of irradiation increased, while a higher dose (40 Gy) decreased, the DA and TA sensitivity of PTTH neurons. We also examined cell viability and morphology of PTTH neurons after exposure to ionizing radiation, which could influence the physiological responses. Neurons that are damaged and dying are strongly depolarized and may exhibit reduced responses to KCl stimulation and neurotransmitters. Morphological changes are thought to accompany physiological alterations. Irradiation of larvae with a 40-Gy dose had harmful effects on the viability and morphology of PTTH neurons, consistent with the reduction of larvae-to-adult survival. In contrast, 25 Gy of gamma irradiation increased the effect of inhibitory (DA and TA) neurotransmission on PTTH neurons, without compromising neuronal viability and survival of flies, which still resulted in delayed pupariation. This tissue-wide radiation exposure might also affect additional timing signals in PG besides PTTH and the interacting neurons in the circuits, which could further delay the development. Further analysis of neurotransmission on PTTH neurons and consequent developmental progression in *Drosophila* could provide a powerful platform for understanding the basic mechanisms of radiation-induced alteration in synaptic signal transduction in the nervous system.

Radiotherapy, including photon therapy (such as gamma and X-ray radiation) and proton therapy, is considered a major treatment for solid tumors. Historically, radiation-induced molecular pathogenesis has been intensively investigated (Kempf et al., 2014; Verreet et al., 2016). Numerous studies have established behavioral paradigms to analyze long-term neurocognitive impairments after photon and proton radiation, including operant conditioning, novel object recognition, and contextual fear conditioning (Britten et al., 2012; Raber et al., 2013; Rabin et al., 2014; Severyukhin et al., 2023). Neuronal activity and synaptic transmission represent important physiological properties, which could be readily measurable and analyzed using calcium imaging and electrophysiological methods in recent years. However, physiological studies of radiation effects on basic neuronal parameters, including intrinsic properties and synaptic parameters, remain relatively rare. These studies have mainly focused on the long-term consequences of proton radiation exposure in the hippocampus, especially the CA1 neuronal network involved in learning and memory (Vlkolinský et al., 2007; Raber et al., 2014; Sokolova et al., 2015; Lee et al., 2017). Hippocampal synaptic plasticity, which is usually determined electrophysiologically as long-term potentiation (LTP), is the principal candidate synaptic mechanism underlying memory formation. Vlkolinský et al. (2007, 2008) showed that high-energy particle (^56^Fe) proton radiation inhibited LTP in mouse CA3-CA1 neuronal circuits. In contrast, low-dose ^28^Si proton radiation enhanced the magnitude of LTP and therefore increased synaptic plasticity in the CA1 region (Raber et al., 2014). Sokolova et al. (2015) reported that proton exposure (1 Gy) hyperpolarized the neuronal resting membrane potential, decreased the input resistance of CA1 pyramidal neurons, and increased rate of miniature excitatory postsynaptic currents. Using electrophysiological and biochemical methods, Lee et al. (2017) demonstrated that low-dose proton radiation (0.5 Gy) increased the inhibitory postsynaptic current in cell type-specific GABAergic neurotransmission in the CA1 region of the mouse hippocampus. It should be noted that all of the above published physiological studies investigated how proton radiation affects functional neurotransmission. High-energy proton radiation causes both displacement and ionization damage, while photon radiation induces only ionizing effects (Goossens et al., 2019). Therefore, photon radiation using gamma sources or X-rays has been widely used to investigate the effect of ionizing radiation in the laboratory. Here, our study shows the first measurement of photon radiation-induced changes in physiological neurotransmitter-evoked responses of postsynaptic neurons. Our results strongly suggest that abnormal responses to neurotransmitters play a significant role in the mechanism of ionizing radiation-induced neurobiological dysfunction.

Our data figure out that gamma irradiation alters DA and TA responses in PTTH neurons. Pharmacological and RNAi experiments demonstrate that TAR1 receptors in PTTH neurons are responsible for TA responses, suggesting they could be affected by gamma irradiation. PTTH neurons integrate information from presynaptic interneurons, primarily composed of seven major sens interneurons, which directly receive inputs from sensory neurons, as well as some non-sens interneurons (Hückesfeld et al., 2021). However, to our knowledge, there are no identified upstream interneurons that provide direct dopaminergic/tyraminergic inputs to PTTH neurons in published works. PTTH neurons could also receive peptidergic inputs from other neurons, such as corazonin (Crz) expressing neurons (Imura et al., 2020). Transcriptome analysis identified that vesicular monoamine transporter (Vmat) was expressed in Crz neurons (Brunet Avalos et al., 2019), suggesting that they could serve as the potential dopaminergic upstream to the PTTH neurons. The dopaminergic/tyraminergic upstream neurons of PTTH neurons and the effect of gamma radiation on their functional connections need to be elucidated in the future. In this study, to optically assess the neuronal activity of single PTTH neurons, GCaMP6m was used to detect changes in somatic calcium concentrations. Several studies have imaged calcium levels while recording electrical activity from the cell body to confirm a direct relationship between somatic calcium signals and action potentials (Hartung and Gold, 2020). However, this indirect measurement is based on the premise that a sufficient number of calcium channels could be regulated when action potentials enter the cell body, driving a detectable change in intracellular calcium concentration. To further explore radiation-induced changes in local synaptic transmission, dendritic and axonal calcium signals would be analyzed using calcium indicators specifically expressed in presynaptic or postsynaptic terminals of PTTH neurons.

Ionizing radiation induces biological effects by directly damaging DNA or indirectly generating ROS, such as superoxide and hydroxyl radicals. ROS are groups of unstable and highly reactive molecules or free radicals responsible for the activation of several stress response effectors, including oxidative stress. Increased ROS production has been recognized as a contributing factor to aging and the progression of several neurological diseases (Tönnies and Trushina, 2017; Doser and Hoerndli, 2021). *In vitro* and *in vivo* studies have shown significantly increased ROS and elevation of ROS-induced oxidative stress following gamma-, X-ray, and proton exposure in animal models (Baluchamy et al., 2012; Baulch et al., 2015; Paithankar et al., 2018). Several studies have demonstrated that ROS play important roles in the regulation of neuronal activity and neurotransmission, under both physiological and pathological conditions. ROS were shown to be involved in maintaining evoked synaptic transmission at neuromuscular junctions of *Drosophila* larvae (Oswald et al., 2018). Sesti et al. (2010) and Han et al. (2017) showed that different types of K^+^ channels could be redox regulated by ROS, which led to loss of neuronal function. ROS could modulate the ion conductance and channel opening probability of GABA type A receptors (Beltrán González et al., 2020). Thiels et al. (2000) demonstrated that increased ROS levels could cause impairments of LTP in the mouse CA1 area and long-term memory. On the basis of these reports, we hypothesize that functional neurotransmitter receptors could be generally susceptible to oxidation and modulated by ROS. Ionizing radiation-induced increases in ROS levels could be critical components of possible mechanisms underlying the altered DA and TA sensitivity observed in our study. Because the ROS elevation in human cells after proton radiation is significantly larger and more rapid than that after photon radiation, it is hypothesized that proton radiation could result in greater effects than photon radiation (Limoli et al., 2007). Thus, whether and how photon radiation affects functional neurotransmission have been insufficiently studied or ignored. In this study, we demonstrated photon radiation-sensitive phenotypes and established a powerful physiological research model for understanding ionizing radiation-induced effects on neurotransmission, which will serve as a starting point for further investigations.

In conclusion, we showed that gamma irradiation alters the DA and TA sensitivity of PTTH neurons in *Drosophila* larvae, revealing for the first time the ionizing radiation-induced changes in functional neurotransmission. DA neurotransmission, including DA synthesis, transport, release, receptors, and signaling pathways in postsynaptic neurons, is highly conserved between *Drosophila* and human (Karam et al., 2020). TA is considered to function analogously to norepinephrine in human (Roeder, 2005). Therefore, it is reasonable to assume that the effects of radiation on cellular receptor signaling in *Drosophila* and human share similar mechanisms. Here, we examined two radiation doses (25 and 40 Gy) within the recommended range for conventional cranial radiotherapy (Hellevik and Martinez-Zubiaurre, 2014; Trifiletti et al., 2020). Our discovery of ionizing radiation-induced alterations in neurotransmitter signaling of PTTH neurons suggests that photon therapy could induce functional neurotransmission abnormalities. Given the important roles that neurotransmission plays in cognitive tasks, this neurotoxic consequence could result in many nervous system disorders after radiation therapy. It is important to explore strategies to mitigate these effects of ionizing radiation in neurotransmission system. One potential approach is to use of antagonists/agonists that bind to neurotransmitter receptors for long-term maintenance of normal synaptic function. For future studies, we aim to identify the upstream neurons that release DA/TA onto PTTH neurons. The effect of ionizing radiation on their functional connectivity could be investigated using calcium imaging combined with optogenetics. On the other hand, we will analyze how ionizing radiation alters dendritic and axonal DA/TA responses of PTTH neurons. These investigations may allow elucidation of the cellular mechanisms underlying radiation-associated neurocognitive dysfunction.

## Data availability statement

The original contributions presented in this study are included in the article/supplementary material, further inquiries can be directed to the corresponding author.

## Author contributions

YiZ, YihZ, LL, and HW designed the research. YiZ, YihZ, CS, SH, WD, and HW performed the research. YiZ, YihZ, and HW analyzed the data and wrote the manuscript. All authors contributed to the article and approved the submitted version.
